# High velocity domain wall propagation using voltage controlled magnetic anisotropy

**DOI:** 10.1038/s41598-019-43843-x

**Published:** 2019-05-14

**Authors:** F. N. Tan, W. L. Gan, C. C. I. Ang, G. D. H. Wong, H. X. Liu, F. Poh, W. S. Lew

**Affiliations:** 1 School of Physical and Mathematical Sciences, Nanyang Technological University, 21 Nanyang Link, Singapore, 637371 Singapore; 2 GLOBALFOUNDRIES Singapore Pte, Ltd., Singapore, 738406 Singapore

**Keywords:** Electronic devices, Magnetic devices

## Abstract

The use of voltage-controlled magnetic anisotropy (VCMA) *via* the creation of a sloped electric field has been hailed as an energy-efficient approach for domain wall (DW) propagation. However, this method suffers from a limitation of the nanowire length which the DW can propagate on. Here, we propose the use of multiplexed gate electrodes to propagate DWs on magnetic nanowires without having any length constraints. The multi-gate electrode configuration is demonstrated using micromagnetic simulations. This allows controllable voltages to be applied to neighboring gate electrodes, generating large strength of magnetic anisotropy gradients along the nanowire, and the results show that DW velocities higher than 300 m/s can be achieved. Analysis of the DW dynamics during propagation reveals that the tilt of the DW and the direction of slanted gate electrode greatly alters the steady state DW propagation. Our results show that chevron-shaped gate electrodes is an effective optimisation that leads to multi-DW propagation with high velocity. Moreover, a repeating series of high-medium-low magnetic anisotropy regions enables a deterministic VCMA-controlled high velocity DW propagation.

## Introduction

Domain wall (DW) memory has been researched upon for many years as it has the potential to be a fast and non-volatile memory device due to the nanosecond timescale of magnetization dynamics, magnetic remanence and high thermal stability^1^. Conventionally, spin torque techniques are used to propagate DWs, such as spin transfer torque and spin orbit torque^1–4^. These techniques often result in stochastic motion of a DW as it is pinned by randomly scattered intrinsic pinning sites that exists in the material. To achieve deterministic pinning, geometric notches and magnetic properties alteration have been used to create energy barriers that acts as pinning sites^1,5–8^. However, a higher excitation energy is required for the DW to depin. Electric field control has also been proposed to pin DWs by locally altering the magnetic anisotropy of the material^9,10^. This technique reduces energy consumption as the pinning site can be turned off when the DW is required to move again.

Spin torque techniques require high current densities which inherently cause resistive loss, resulting in Joule heating. To circumvent the power loss and prevent Joule heating, electric field control methods can be used to manipulate the properties of magnetic devices due to its energy efficient nature^11–13^. Voltage controlled magnetic anisotropy (VCMA) is a form of electric field control that has been demonstrated to reduce the current density required for switching in MTJ devices^14,15^. For DW devices, the energy of a DW can be expressed as *σ*, where $\sigma \propto \sqrt{A{K}_{u}}$. Here, *A* represents exchange stiffness parameter and *K*_*u*_ represents magnetic anisotropy energy. Therefore, DW energy can be reduced by lowering the *K*_*u*_ of the magnetic material through VCMA. The VCMA effect has also been experimentally realized to assist in the propagation of DWs in the creep regime^12^.

**Figure 1:**
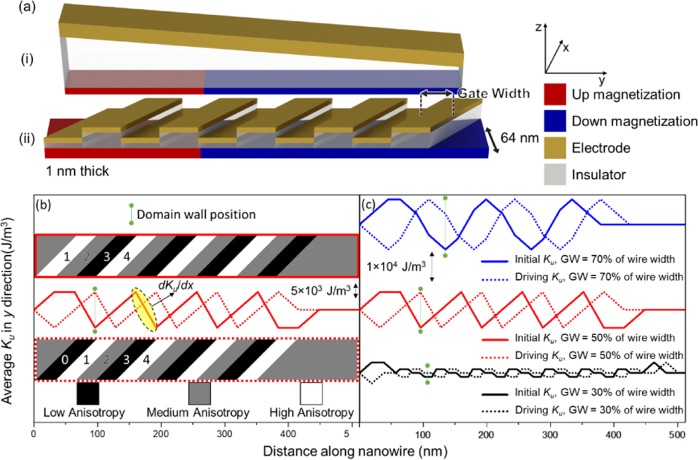
(**a**)(i) Schematic diagram of a sloped insulator to propagate a DW. (**a**)(ii) Proposed schematic structure of slanted gate electrodes to propagate DWs. The magnetic NW is 64 nm in width and 1 nm thick. (**b**) The solid line represents the initial average *K*_*u*_ in the *y* axis. The dotted line represents the average *K*_*u*_ that drives the DW represented by the green vertical line. The top and bottom inset represents the *K*_*u*_ of the NW in the initial and driving stage respectively. The yellow highlighted region represents the gradient of the slope where a linear fit gives the $d{K}_{u}/dx$ that propagates the DW. (**c**) A comparison of the various GW as a percentage of the NW width, where 30/50/70% of the NW width equates to a GW of 20/32/44 nm. An anisotropy change of +20 kJ/m^3^ and −20 kJ/m^3^ was applied to the high and low anisotropy regions respectively to illustrate the *K*_*u*_ dependence across the length of the NW.

A force exerting on the DW in the *x* direction can be described using the DW energy and *K*_*u*_ of the magnetic nanowire (NW), $F(x)=-\,(\partial \sigma /\partial {K}_{u})\cdot (\partial {K}_{u}/\partial x)$. Hence, a magnetic anisotropy gradient in the *x* direction, along the NW ($d{K}_{u}/dx$) will exert a force on the DW. By using a wedged insulator as shown schematically in Fig. 1(a)(i), a sloped electric field can be generated, giving rise to a $d{K}_{u}/dx$ which can be used to propagate or assist in the propagation of a DW^1,9,16–24^. However, such a wedge structure does not scale well for a longer NW, as the lengthened NW would result in increasingly thicker or thinner insulators at the ends of the NW. At the end with the thinner insulating layer, the strong electric fields may lead to a dielectric breakdown of the insulating layer. Furthermore, it is recently reported in MTJs that the VCMA coefficient is only linear at low electric field but saturates at higher electric field^25,26^. This poses a limitation on the length of the NW for a sloped VCMA DW memory device.

In this work, we demonstrate an energy efficient DW propagation technique whereby a $d{K}_{u}/dx$ is created along a NW with the use of a repeating series of gate electrodes. Three of the gate electrodes can create local high-medium-low *K*_*u*_ region which can be used to propagate DWs with deterministic displacements at high velocities on a NW of any length. The DW dynamics due to the manipulation of *K*_*u*_ using the gate electrodes are analysed, leading towards an optimized DW propagation technique. The results provide a promising path towards high velocity and accurate DW memory device using VCMA.

## Results

A series of gate electrode is used to create a $d{K}_{u}/dx$, this is similar to the proposed techniques of VCMA gradient skyrmion memory device^27,28^. Unlike a skyrmion which can be ~50 nm in diameter. The width of a DW in a material with perpendicular magnetic anisotropy is usually ~10 nm. Using the same technique proposed for skyrmion propagation for DWs would require a gate electrode of ~3 nm to propagate DWs. This poses significant technical challenges for experimental realization. Therefore, slanted gate electrodes are proposed to overcome this challenge and provide a smooth average $d{K}_{u}/dx$ as schematically shown in Fig. 1(a)(ii). The insulating layers have alternating thickness to prevent electrical contact between the gate electrodes, it is important to note that the insulating layer thickness does not change during DW propagation, only the gate voltage applied. Although a high-medium-low *K*_*u*_ region is desired, a two-thickness insulator variation model is used instead of a three different insulator thicknesses. This is detailed in the supplementary material by Fig. S1.

A repeating series of $d{K}_{u}/dx$ is created when the adjacent regions of the NW have different *K*_*u*_ due to the modulation of the VCMA effect. A repeating series of three gate electrodes results in a high-medium-low *K*_*u*_ region as shown in the upper diagram (solid border) in Fig. 1(b), where the high-medium-low region is labelled as 1, 2 and 3 respectively. In this instance, the DW energy is at a minimum when the DW is at the low *K*_*u*_ region. By switching the gate voltage to shift the *K*_*u*_ region of the NW towards the right, as shown in the lower diagram (dotted border), the position of lowest energy is shifted by one gate electrode length. This results in a $d{K}_{u}/dx$ experienced by the DW which cause it to be propagated along the NW. The low *K*_*u*_ region sandwiched between the high *K*_*u*_ regions forms a potential well, which allows the DW to move deterministically. The probability of the DW crossing the high *Ku* regions at room temperature can be calculated using $P=\frac{{e}^{-{\sigma }_{high}/{k}_{b}T}}{{e}^{-{\sigma }_{high}/{k}_{b}T}+{e}^{-{\sigma }_{low}/{k}_{b}T}}$, where *P* is the probability, *k*_*b*_ is the Boltzmann constant, *T* is the temperature and *σ* is the DW energy. An anisotropy change of +40 kJ/m^3^ and −40 kJ/m^3^ for the high and low anisotropy region corresponds to a $d{K}_{u}/dx$ of 625 GJ/m^4^. The calculated probability from these values is ~1 × 10^−8^, which is similar to the write error rate for NAND flash and MTJ devices. However, the high *K*_*u*_ region which ensures deterministic motion also introduces undesired DW dynamics during propagation that will be discussed later in this report.

Figure 1(c) shows the *K*_*u*_ along the NW with the gate width (GW) as a percentage of the width of the NW. The DW position illustrated in Fig. 1(b,c) schematically shows where the position of the DW would be before the gate voltages are changed. As shown in Fig. 1(c), to create a uniform $d{K}_{u}/dx$, the GW is designed to be half of the NW width. Furthermore, when GW is half of the NW width, it produces the highest $d{K}_{u}/dx$, ideal for high speed DW propagation. This is shown in Fig. S2 from the supplementary materials, where the average $d{K}_{u}/dx$ in the *x* direction with varying gate width is shown. Therefore, the study of the propagation of DW under a $d{K}_{u}/dx$ in this work will be utilizing a GW of 32 nm as the NW width used is 64 nm.

**Figure 2:**
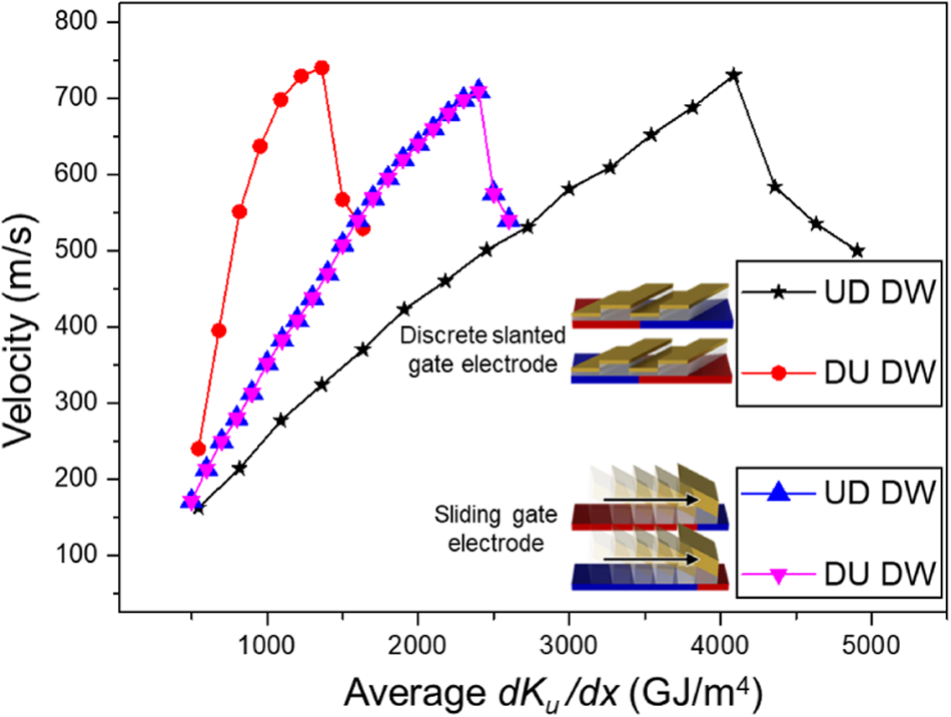
DW velocity of UD and DU DW propagated by the slanted gate electrodes is plotted against $d{K}_{u}/dx$. The DW velocity from a dynamically sliding $d{K}_{u}/dx$ configuration is also shown for comparison. The insets show the schematic diagram for both the structures.

To study the DW dynamics during propagation under the VCMA effect, the center of the DW is taken to be its position, and a continuous $d{K}_{u}/dx$ induced by a moving wedge electrode that follows the DW position. In the case of discrete gate electrodes, the gate voltages switches once the DW reaches the point of lowest *K*_*u*_. This allows the high-medium-low *K*_*u*_ region created by the gate voltage to follow the position of the DW in a discrete manner. The $d{K}_{u}/dx$ is obtained by a linear fit of the driving anisotropy gradient as highlighted in Fig. 1(b). Figure 2 show a down-up (DU) and up-down (UD) DW propagated by either discrete slanted gate electrodes or a moving wedge electrode. The moving wedge creates a continuous $d{K}_{u}/dx$ that slides along with the DW as it is propagated and acts as a control to illustrate the DW dynamics caused by the slanted gate electrodes. The abrupt decrease in velocity with increasing $d{K}_{u}/dx$ in all the configurations shown in Fig. 2 is due to the well-studied Walker breakdown phenomenon^19,29,30^. The Walker breakdown for a DW propagated with a sliding gate electrode and propagation by a magnetic field is phenomenongically similar and is shown in the Fig. S3 of the supplementary material. Using the discrete slanted gate electrodes, there is a difference in steady state velocity of the UD and DU DW even though the same $d{K}_{u}/dx$ was applied on the DW. However, this was not observed when the DW is propagated by the continuous sliding $d{K}_{u}/dx$. The difference in velocities may be a consequence of the DW tilt which is caused by the Dzyaloshinskii-Moriya interaction (DMI) of the NW^31–33^. The DMI of the NW in this comparison between UD and DU DW is 2 mJ/m^2^.

**Figure 3:**
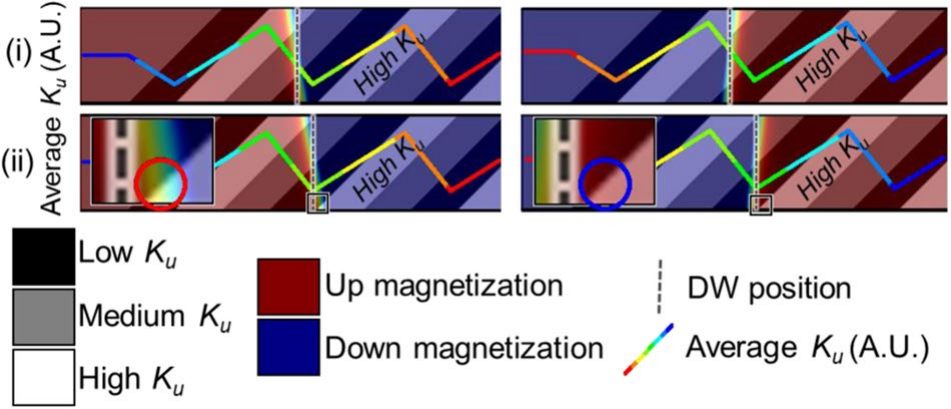
A comparison of a UD and DU DW being propagated using slanted gate electrodes in from (i) to (ii). The averaged *K*_*u*_ along the *y* direction is superimposed onto the NW as a rainbow line plot for visual aid (i) The DWs moves towards the right under the effect of the $d{K}_{u}/dx$. (ii) The DW reaches the lowest averaged *K*_*u*_ in the *x* direction, highlighting the encroachment caused by the high *K*_*u*_ region in the UD DW and lack thereof in the DU DW.

Figure 3 shows the DW magnetization superimposed on the *K*_*u*_ region of the NW. The averaged *K*_*u*_ along the *x* direction and the center position of the DW is also shown. Both UD and DU DWs are shown on the left and right of the figure respectively. In Fig. 3(i) the DW experiences a $d{K}_{u}/dx$. This cause the DW to move rightwards as illustrated in Fig. 3(ii) where the bottom of the UD DW tilts towards the high *Ku* region. The red circle in the enlarged image, highlights the DW clearly encroaching into the high *K*_*u*_ region. However, this encroachment is not present in the DU DW as shown in the blue circle of the enlarged image. This difference is caused by the mismatch between the DW tilt and gate electrode slant angle. For the UD DW, the encroachment of the DW caused by the DW tilt angle mismatch is found to occur after the DW moves ~16 nm after the gate electrodes switches. This results in a periodical change in velocity after the DW moves ~16 nm, resulting in the spectral analysis presented in Fig. S4 of the supplementary material. Due to the NW symmetry, a UD DW moving to the right is equivalent to a DU DW moving to the left. Hence, only the motion to the right is reported for conciseness.

While it is feasible to propagate a single DW using the slanted gate electrodes, the difference in velocity of the UD and DU DWs will result in a multi-DW propagation to be limited by the slower DW. To prevent this asymmetry of DW propagation, the gate electrode shape should be symmetrical along the nanowire axis. As such, the use of chevron-shaped gate electrodes to propagate the DWs was investigated. The chevron gate electrodes were designed to have a steeper angle of 31° compared to 45° for the slanted gate electrodes so that the average $d{K}_{u}/dx$ for both systems are similar, as shown in Fig. S5 of the supplementary material.

**Figure 4:**
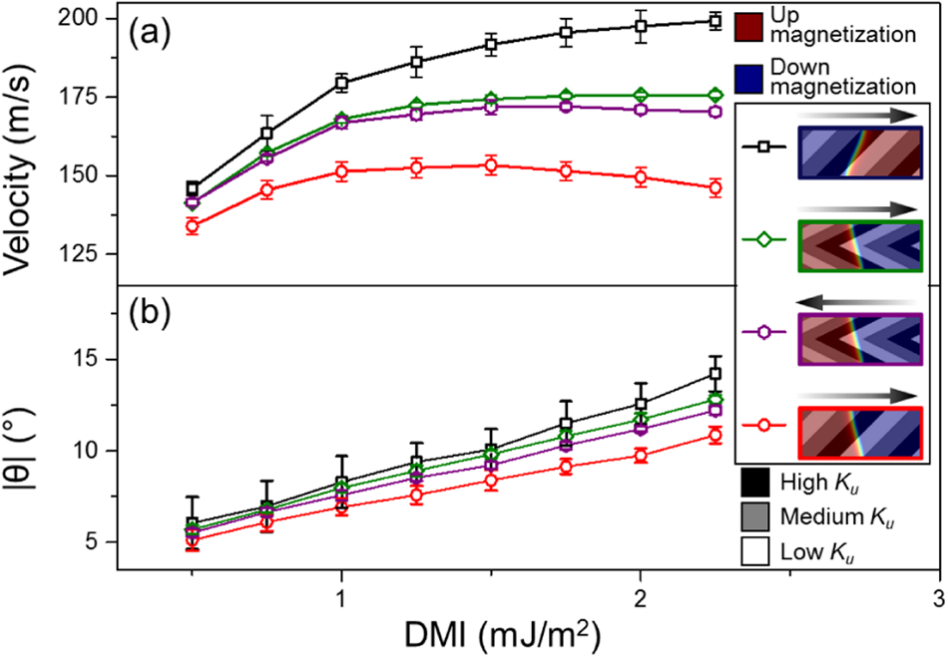
The legend shows the superposition of the DW magnetization with the *K*_*u*_ regions, illustrating the configurations of DW and gate electrodes. The arrow represents the direction of propagation for the DW. The DWs in this figure are propagated by a $d{K}_{u}/dx$ of 625 GJ/m^4^. (**a**) Steady state DW velocity of the various configurations with varying DMI. (**b**) The angle of the DW while in steady state propagation with varying DMI.

The tilt of the DW in steady state was varied by changing the DMI of the magnetic material^19,31,32^. Figure 4(a,b) shows the steady state DW velocity and tilt respectively for DWs propagated using chevron and slanted gate electrodes. For slanted gate electrodes, the results in Fig. 4 confirm that the increase in DW tilt angle mismatch results in a lower DW velocity. As the DMI increases, the difference in velocity and DW tilt between the two configurations increases, indicating that the slower velocity is due to the DW tilt angle mismatch.

Due the axial symmetry of the chevron gate electrodes, there is no difference in steady state DW velocity and DW tilt for a DU or UD DW during steady state propagation travelling in the same direction. However, as the chevron shape has lateral asymmetry, the steady state of the DW moving in opposite direction differs. As the DW moves towards the left, the DW tilt angle mismatch illustrated in Fig. 3 happens for both UD and DU DW due to the high *K*_*u*_ region at both the upper and lower edge of the NW. The steeper angle of the chevron gate electrode reduces the extent of which the DW encroaches into the high *K*_*u*_ region as compared to using slanted gate electrodes. This results in different DW velocity and DW tilt for the slanted and chevron gate electrodes shown in Fig. 4. When the chevron gate electrodes are used to propagate the DW rightwards, the higher *K*_*u*_ region is at the middle of the NW, hence, the tilt of DW does not increase the DW encroachment into the higher *K*_*u*_ region. This results in the higher DW velocity for DWs moving towards the right than moving towards the left.

Since UD and DU DWs need to propagate at similar velocities when travelling in the same direction to avoid write errors. A NW containing multiple DWs needs to be propagated by chevron gate electrodes. This is illustrated in Video S6 of the supplementary material, where two DWs in a nanowire are propagated with a driving frequency using chevron and slanted gate electrodes. The domain propagated using chevron gate electrode does not suffer from any deformation, whereas the domain propagated using the slanted gate electrodes becomes larger or stops moving due to the difference in velocity between the leading and trailing DW. As the DWs move faster towards the right than the towards left, a mono-directional DW device can be employed for faster DW device operation. The magnetization bits can be stored as a delay line memory or looped into a circle much like a magnetic platter in HDD.

Figure S7 in the supplementary material shows the DW velocities dependence on $d{K}_{u}/dx$. The DWs were propagated using chevron gate electrodes with a DMI value of 1 mJ/m^2^ to match experimental CoFeB results^34^. DW velocity of up to 316 m/s is achieved before Walker breakdown occurs. This is comparable to the DW velocities achieved using spin torque techniques^2,4,35^. The $d{K}_{u}/dx$ used to achieve 316 m/s is 975 GJ/m^4^, which corresponds to an anisotropy change of 62 kJ/m^3^. As VCMA coefficient has been reported to be >290 fJ/Vm^36,37^, the anisotropy change of ±62 kJ/m^3^ can be easily achieved with an applied electric field of 0.2 GV/m, which is the range of the electric field used on magnetic magnetics for electric field control study^11,38,39^.

**Figure 5:**
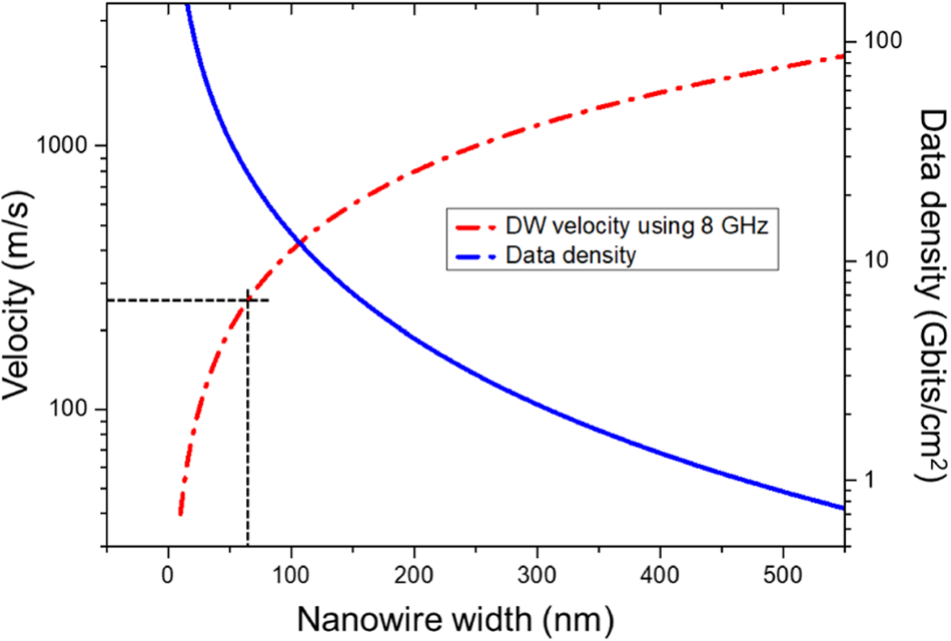
Relation between the DW velocity and data density with varying nanowire width. The *y* axes are in log scale for better visual representation. The black dotted lines represent the 64 nm NW width used in this work, corresponding to a maximum DW velocity of 256 m/s with a clock speed of 8 GHz.

Apart from Walker breakdown, another limiting factor of the DW velocity is the frequency at which the gate voltage can be changed. Assuming a clock speed of 8 GHz, a steady state DW velocity of 256 m/s can be achieved, and ~9.9 GHz is required to reach 316 m/s using a 32 nm gate width on a 64 nm-width nanowire. A larger gate and nanowire width will result in a higher velocity as the DW would move across the longer gate width per cycle. However, increasing the gate and nanowire width comes at the cost of data density. A relationship between the DW velocity and data density using the proposed model is as shown in Fig. 5.

In conclusion, a comprehensive study on the viability of slanted and chevron-shaped gate electrodes to propagate DW at high velocities was conducted. The discretized DW movement induced by the array of gate electrodes confines DW to precise locations, thus removing the need to create permanent pinning sites. With slanted gate electrodes, the DW tilt angle mismatch results in the velocity difference between UD and DU DW configurations. When using the axially symmetrical chevron gate electrodes, all DWs are propagated at the same velocity on the NW, achieving high DW velocities of up to 316 m/s, which is ~6 times faster than a DW propagated by a slope electric field^19^. The results presented in this work paves the way for energy efficient high velocity DW devices using the VCMA effect.

## Methods

The micromagnetic simulations are performed using MuMax3. MuMax3 solves the Landau–Lifshitz–Gilbert equation in discrete time steps using $\frac{\overrightarrow{dM}}{dt}=-\,\gamma {\overrightarrow{H}}_{eff}\times \overrightarrow{M}+\frac{\alpha }{{M}_{s}}\overrightarrow{M}\times \frac{d\overrightarrow{M}}{dt}$, where γ is the gyromagnetic ratio, α is the damping parameter, *M* is the magnetization, and *H*_*eff*_ is the effective field which includes the *K*_*u*_ energy and interfacial DMI. The work is done using a mesh of 1 nm × 1 nm × 1 nm, which is sufficiently smaller than the exchange length and the DW width. The magnetic material parameters are meant to simulate a CoFeB/MgO-based structure and are as follows: saturation magnetization M_s_ = 8 × 10^5^ A/m, exchange stiffness = 22 × 10^−12^ J/m^2^, *K*_*u*_ = 0.8 × 10^6^ J/m^3^ and the Gilbert damping coefficient = 0.03.

## Supplementary information

Supplementary info

Supplementary video
