# Author Correction: High velocity domain wall propagation using voltage controlled magnetic anisotropy

**DOI:** 10.1038/s41598-019-54255-2

**Published:** 2019-11-21

**Authors:** F. N. Tan, W. L. Gan, C. C. I. Ang, G. D. H. Wong, H. X. Liu, F. Poh, W. S. Lew

**Affiliations:** 1 School of Physical and Mathematical Sciences, Nanyang Technological University, 21 Nanyang Link, Singapore, 637371 Singapore; 2 GLOBALFOUNDRIES Singapore Pte, Ltd., Singapore, 738406 Singapore

Correction to: *Scientific Reports* 10.1038/s41598-019-43843-x, published online 14 May 2019

A supplementary video file was omitted from the original version of this Article. This has been corrected in the HTML version of the Article; the PDF version was correct at the time of publication.

