# Supplementary material for: High velocity domain wall propagation using voltage controlled magnetic anisotropy
Source: Sci Rep. 2019 May 14;9:7369. doi: 10.1038/s41598-019-43843-x (PMC6517393; doi:10.1038/s41598-019-43843-x)
Supplement: Supplementary file 1 — Supplementary info [file 41598_2019_43843_MOESM1_ESM.docx]

Supplementary Information for

“**High velocity domain wall propagation using voltage controlled magnetic anisotropy**”

F.N. Tan^1,2^, W.L. Gan^1^, C.C.I. Ang^1^, G.D.H. Wong^1^, H.X. Liu^2^, F. Poh^2^, and W.S. Lew^1, a^

*^1^ School of Physical and Mathematical Sciences, Nanyang Technological University,*

*21 Nanyang Link, Singapore 637371, Singapore*

*^2^ GLOBALFOUNDRIES Singapore Pte, Ltd., Singapore, 738406*


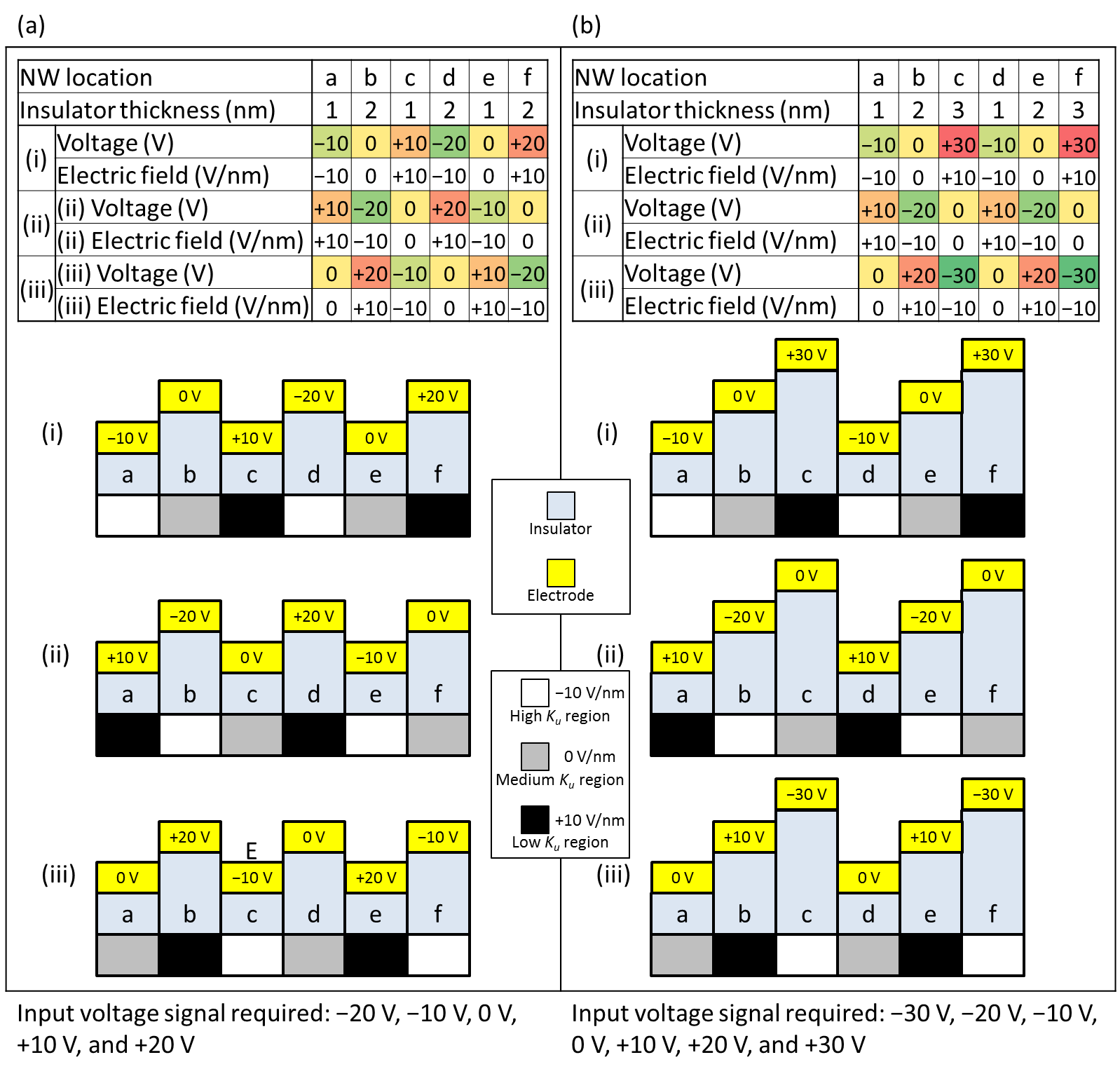


Figure S1. Schematic illustration of the voltages required to shift the anisotropy profile along the NW with (a) two varying insulator thicknesses and (b) three varying insulator thicknesses.


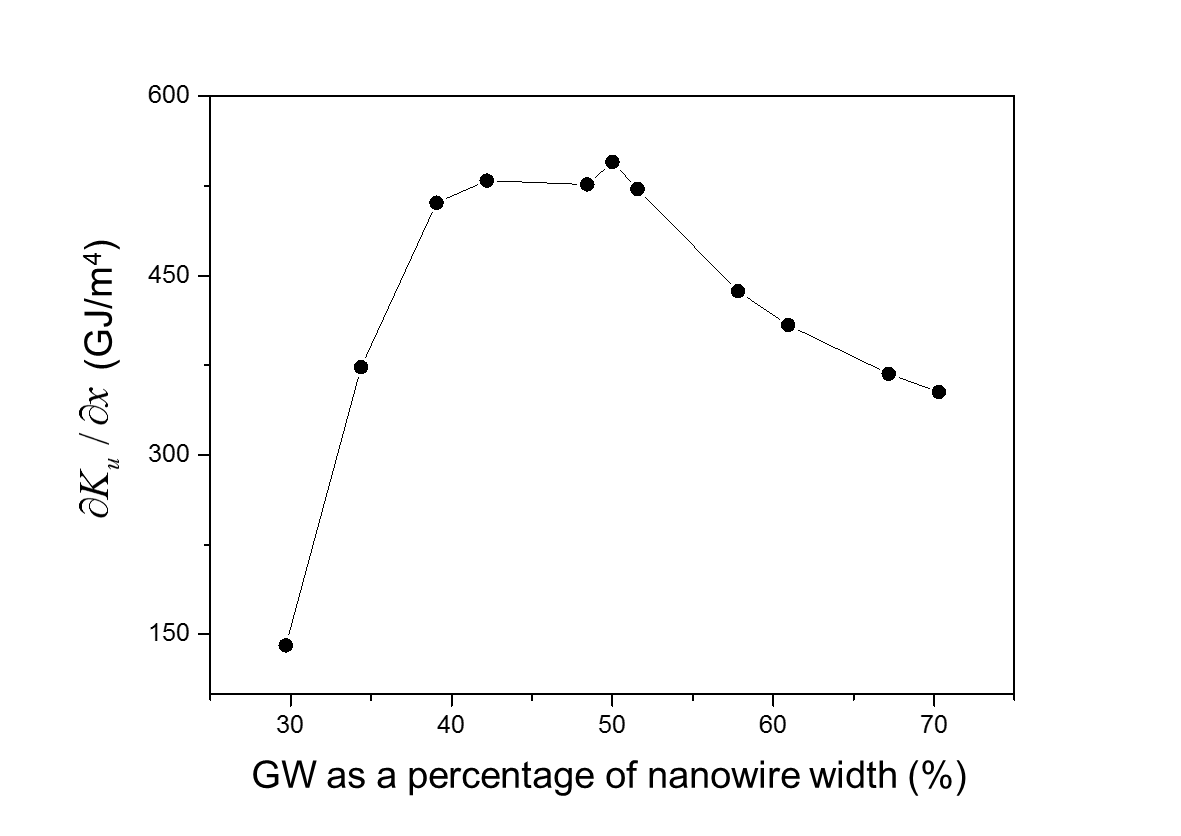

Figure S2. The low and high *K_u_* region used to obtain the $dK_{u}/dx$ is ±0.2% of the nanowire *K_u_*. The largest $dK_{u}/dx$ occurs when the GW is 50% of the nanowire width. Hence, for most of the work in the main text, a GW of 50% is used unless otherwise stated.


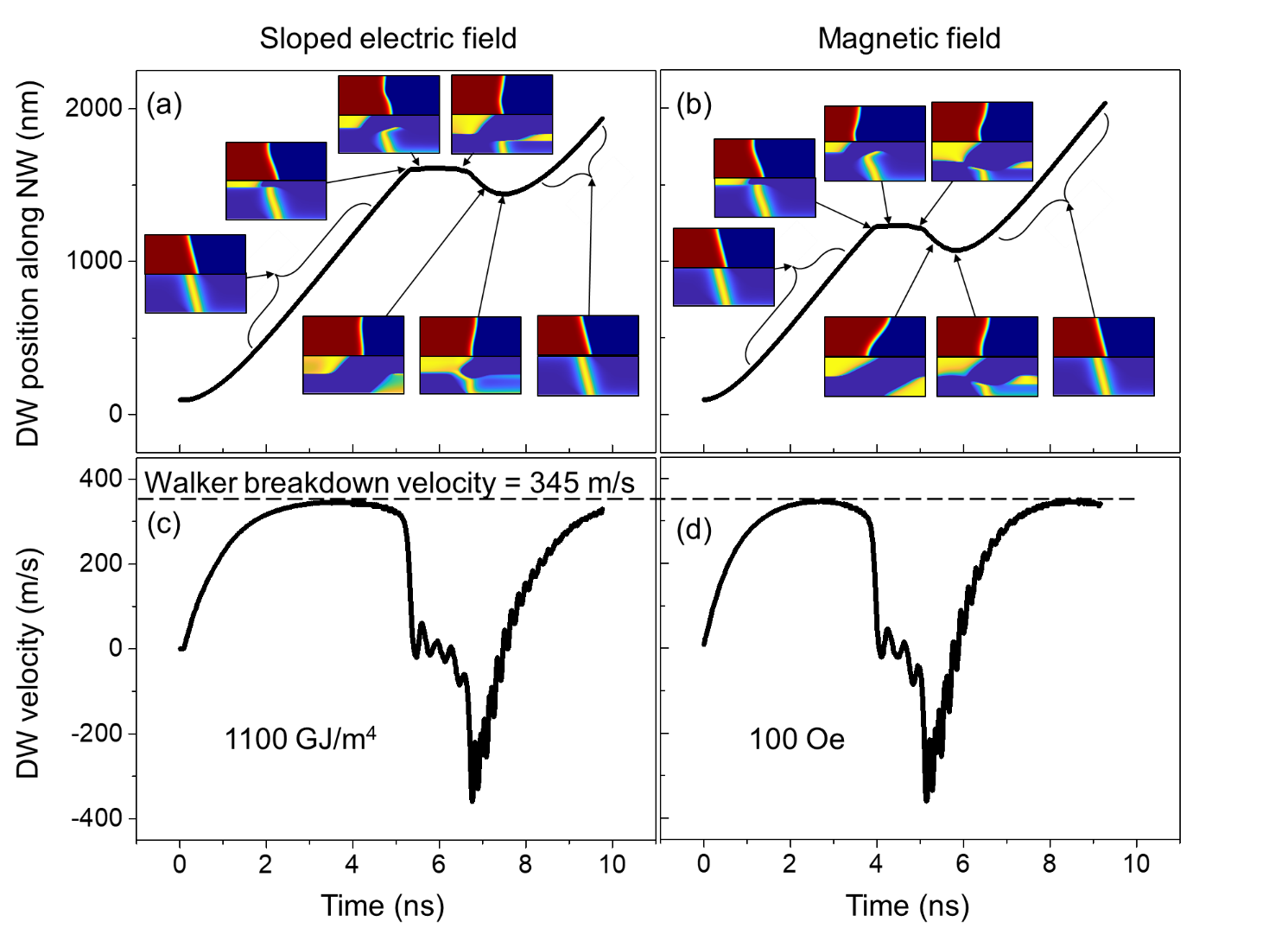


Figure S3. (a) and (b) shows the DW position along the NW for DW propagated by a dynamically sliding $dK_{u}/dx$ and magnetic field respectively. The insets show the magnetization of the DW at the highlighted regions/points along the NW where the top half of the inset shows the out-of-plane magnetization and the bottom half shows the in-plane magnetization. (c) and (d) shows the corresponding DW velocity for (a) and (b) respectively. Both propagation methods show similar results and have a Walker breakdown velocity of 345 m/s. The DMI of the material for these results is 1 mJ/m^2^.


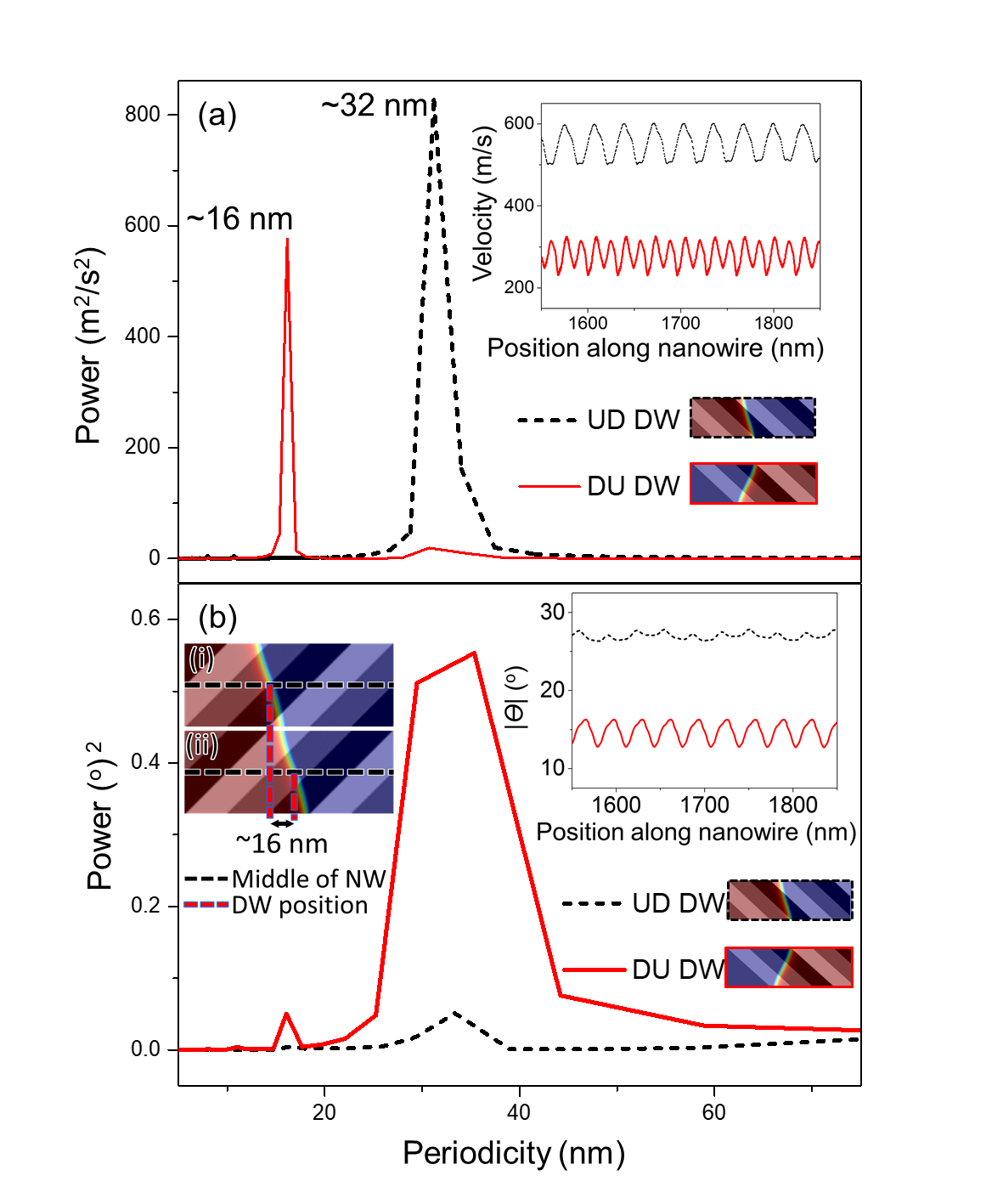


Figure S4. The red solid(black dotted) line represents the DU(UD) configuration where the tilt of the DW is in the opposite(same) direction of the slant of the gate electrodes. The main figures show the fast Fourier transform of their respective inset on the right. The periodicity peaks in the motion of the DW indicate a cyclical nature every 32 nm DW moves due to the switching gates. However, in the DU DW, where the tilt of the DW and the slant of the gate electrodes are in opposite directions, there is an additional periodicity at ~16 nm caused by the encroachment as shown in the main article. This is reflected both in the velocity of the DW as shown in (a) and the tilt of DW as shown in (b). In (b)(i), the DW reaches the lowest *Ku* region before the gate switches. The DW propagates after the gate switches, as shown in (b)(ii) and the encroachment into the high Ku region begins after the DW moves 16 nm across the NW.


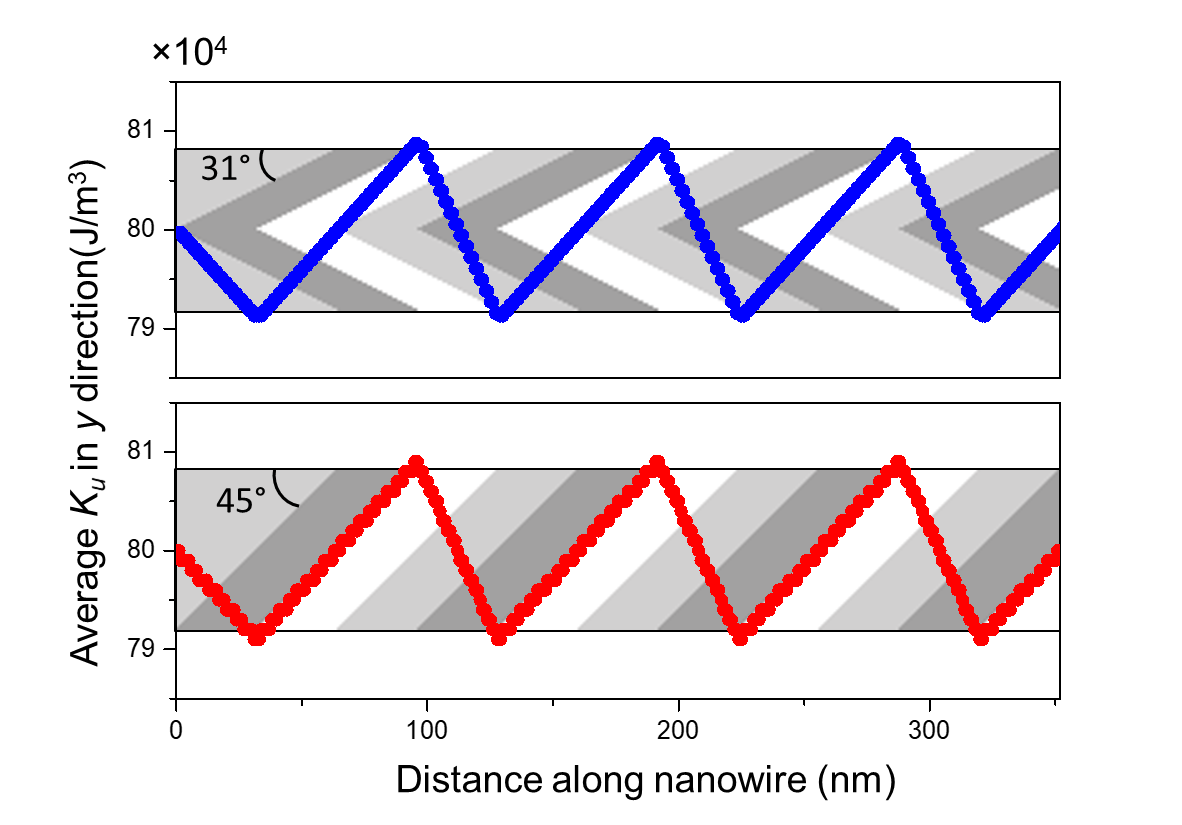


Figure S5. Average *K_u_* in the *x* direction of the NW superimposed with the *K_u_* region of the NW. To maintain the same average *K_u_* across the NW as the slanted gate electrode. The chevron-shaped gate electrodes have a steeper angle to elongate the Ku region across the NW to replicate the average *K_u_*.

Video S6. Chevron and slanted gate electrodes propagating two DWs along the NWs with frequency accelerated towards 8 GHz. The NWs are 2048 nm in length and 64 nm in width. The domain propagated by chevron gate electrodes does not result in any deformation apart from the DW tilt. However, for the slanted electrodes, the DW tilt angle mismatch causes one of the DW to be slower. In the middle NW, the domain elongates as the trailing DW is unable to catch up with the frequency of the switching gate electrode. In the bottom NW, the leading DW is the one unable to catch up with the frequency of the switching gate electrode, which blocks the propagation of the trailing DW. As the trailing DW approaches the leading DW, DW-DW repulsion occurs as shown at ~ 1 ns of simulation time in the video.


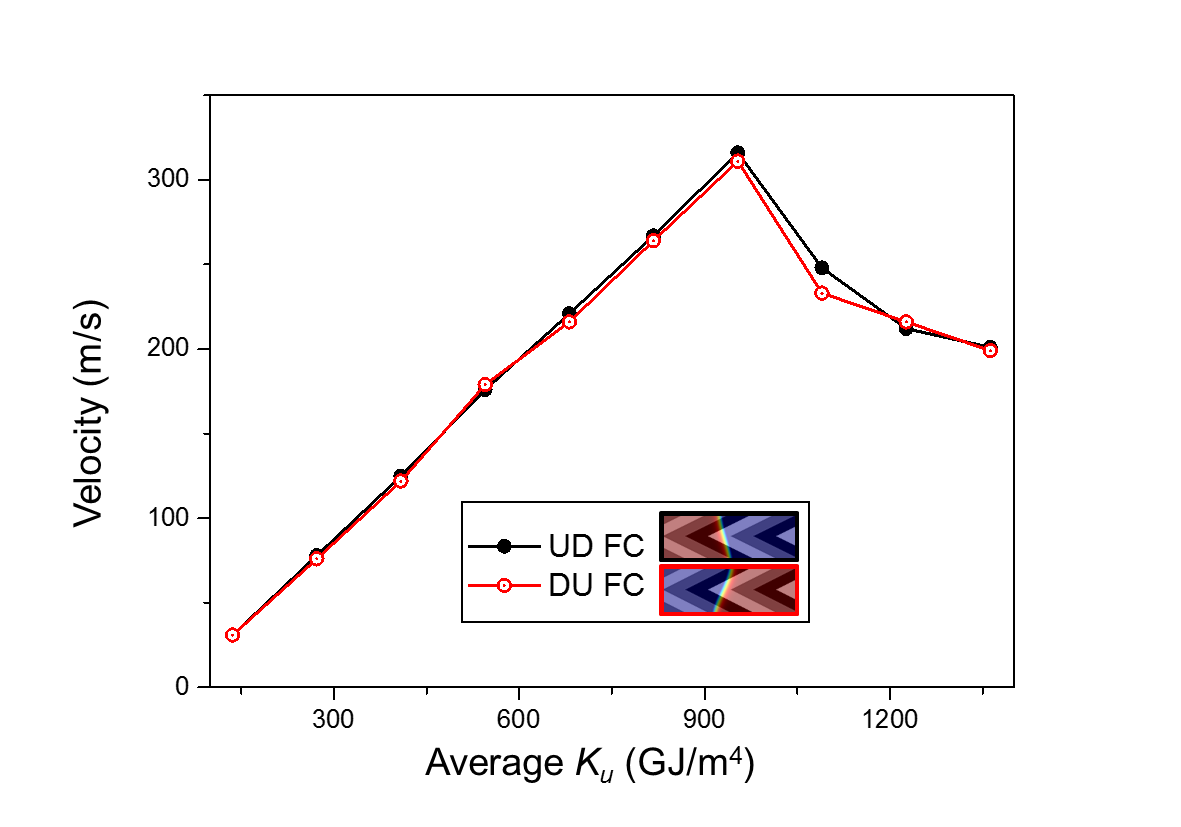


Figure S7. Velocity of DW in with FC gate electrodes. The UD and DU DWs have similar velocities and can reach up to ~316 m/s. The DMI is chosen to be 1 mJ/m^2^, matching experimental results from CoFeB based PMA material.
